# A Rare Case of Conjoined Twins

**DOI:** 10.7759/cureus.48289

**Published:** 2023-11-05

**Authors:** Shabista Shaikh, Pooja Biradar, Amey Chugh, Nikita Bhattacharjee, Tanvi Nijhawan

**Affiliations:** 1 Obstetrics and Gynaecology, Dr. D. Y. Patil Medical College, Hospital & Research Centre, Pune, IND

**Keywords:** twin pregnancy, termination of pregnancy, siamese twins, omphalophagus, conjoined twins

## Abstract

Identical twins joined in utero are called conjoined twins. They are also called “Siamese twins.” It is one of the uncommon variants of twin pregnancy. Our case report aims to demonstrate the significance of prenatal diagnosis and evaluation of conjoined twins due to the increased risk of perinatal morbidity and death. Early prenatal diagnosis and assessment of the degree of joining provide an opportunity for parents to decide whether to continue the pregnancy.

## Introduction

One of the uncommon types of twin pregnancy is conjoined twin pregnancy, which accounts for 1:50,000 to 1:100,000 live births [1]. In Southeast Asia and Africa, the incidence is higher. One-third of conjoined twin babies pass away within a day, and half of them are stillborn. Females are in majority with a ratio of 3:1 (female:male). Although the pathogenesis of this malformation is unknown, it is thought that monozygotic twin pregnancies are caused by an abnormal division of a single zygote (fission theory) after 13-15 days of embryonic period or, more likely, by the secondary association of two distinct embryonic discs (fusion theory) [2]. Twins that are conjoined have the same chorion, placenta, and amniotic sac.

Spencer was the first to categorize and name conjoined twins based on the most obvious point of fusion [3]. The ventral, dorsal, or lateral groups are possible locations for the conjoined site. Conjoined twins are categorized into the following groups based on the location of shared anatomy: thoracopagus (42%) (most common), parapagus dicephalus (11.6%), cephalopagus (5.5%), omphalopagus (5.5%), parasitic (3.9%), craniopagus (3.4%), parapagus diprosopus (2.9%), ischiopagus (1.8%), rachipagus (1%), pygopagus (1%), and unspecified (21.4%) [4].

Due to the increased risk of neonatal morbidity and mortality, conjoined twins should be monitored prenatally by ultrasonography in the first trimester. Ultrasound scanning and magnetic resonance imaging can be utilized to confirm and clarify the diagnosis in the second trimester by establishing the precise anatomy of the two fetuses and the parts fused in detail [5,6]. Ultrasound features that encourage the identification of conjoined twins include inseparable body and skin contours, fetuses facing one another with hyperflexed cervical spines, fewer limbs than expected, organ sharing, and a single umbilical cord with more than three veins [7,8].

Depending on the body parts shared, the prognosis of the conjoined twin differs. Up to 60% of twins die in utero; 35% are stillborn or die within the first 24 hours of delivery [9]. An estimated 70%-75% of instances involve female fetuses, and it is described as a sporadic condition with no known associations [10].

In order to execute a surgical division of conjoined twins, anatomical information is necessary, and this information can only be provided by diagnostic imaging tools. After the confirmation of the diagnosis, accurate family counseling is essential to discuss the various treatment options, which may include pregnancy termination, selective feticide, and postpartum surgical twin separation. We describe an extremely rare case of early second-trimester pregnancy-diagnosed omphalopagus conjoined twins with a shared heart.

## Case presentation

**Figure 1:**
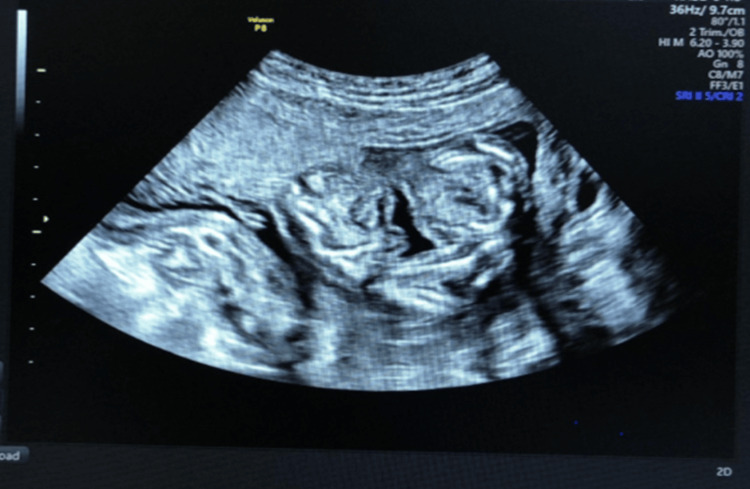
Two-dimensional ultrasound image

A 25-year-old multigravida female with 17 weeks of gestation was referred to the obstetrics and gynecology department. The patient had no abnormal or significant medical history and was in good overall health. She had no personal or family history of twins. On meticulous ultrasound examination, it was revealed that this was a monochorionic, mono-amniotic conjoined twin gestation with growth disparity. They were conjoined at the ventral surface, which is termed as omphalopagus. The smaller twin was 14 weeks in size and showed findings suggestive of enlarged bilateral lateral ventricles with cortical mantle thinning and an enlarged third ventricle suggestive of aqueduct stenosis and tetra-amelia (i.e., agenesis of all four limbs). The larger twins showed growth commensurate with the period of gestation according to the last menstrual period, i.e., 17 weeks in size. No intervening membrane could be seen between the two fetuses, and they showed variable lie. A single shared heart could be well appreciated. Amniotic fluid was adequate for gestational age. The conjoined twins demonstrated active translator movements as a single entity, and the limb movements of the larger twin were distinctly appreciated; the torso movements of the smaller twin were also noted (Figure 1).

**Figure 2:**
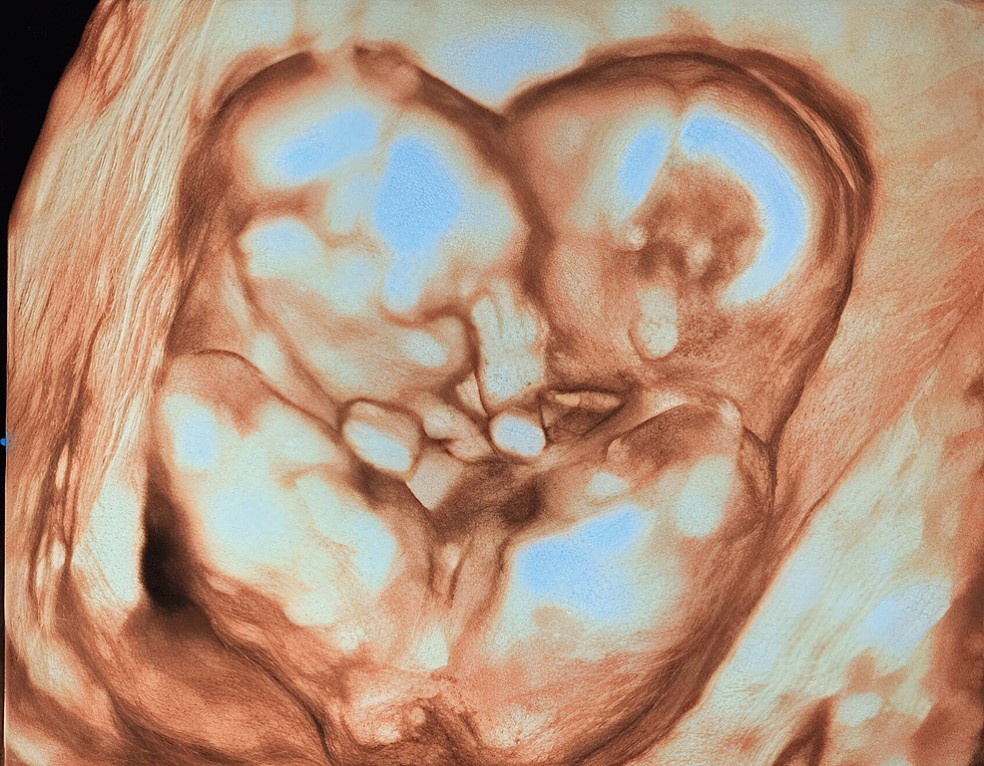
Volume-rendered three-dimensional ultrasound image of the twins showing that the twins are facing each other and appear to be fused on their ventral surface at the level of the abdomen

Conjoined omphalopagus twins were determined by a transvaginal ultrasound examination, a sagittal scan of the thorax, and an abdominal scan. The three-dimensional ultrasound scan confirmed the diagnosis and revealed twins joined at the ventral surface, fusion appeared to be at the level of the abdomen, and there is a high index of suspicion in such fetuses that they may be conjoined by the bowel (Figure 2).

Based on these ﬁndings, the diagnosis of omphalopagus conjoined twins was made, with twin A being 14 weeks with tetra-amelia (absence of all four limbs) and aqueductal stenosis and twin B being 17 weeks with normal gestation.

**Figure 3:**
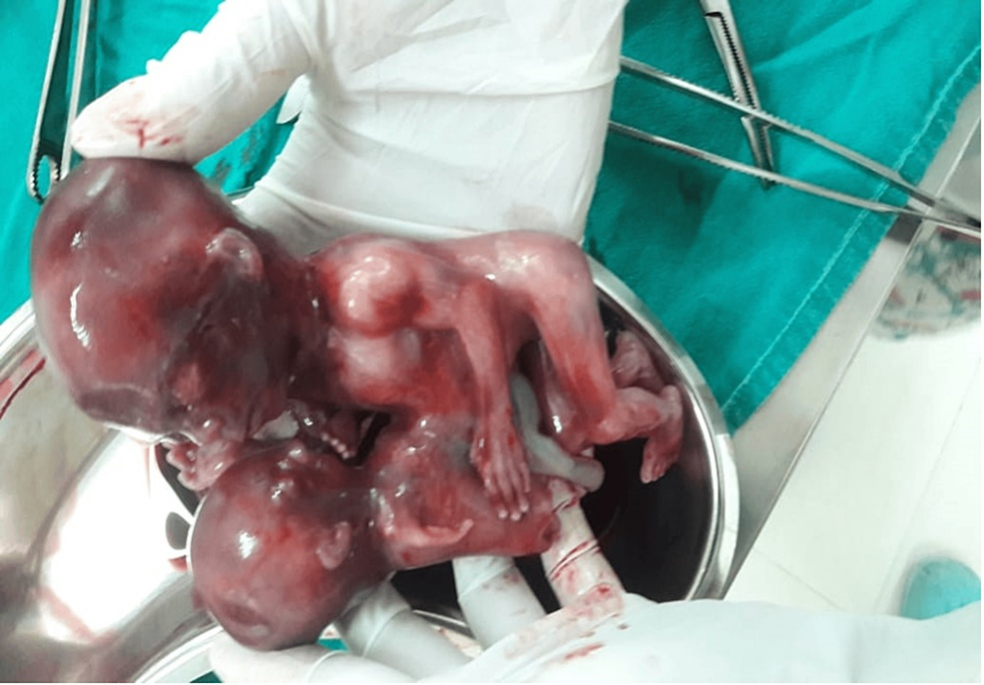
Intact omphalopagus conjoined twins after abortion

After diagnosis, group counseling by pediatricians and gynecologists was done by illustrating all possible options. Parents and relatives were explained about the malformation, poor survival rate, and the possibility of neurological deficit of the surviving twins, and they opted for termination of pregnancy. Consent for second-trimester medical termination of pregnancy (MTP) was taken. The patient was induced for second-trimester abortion as per schedule. She was given an oral tablet mifepristone 200 mg, followed by an oral tablet misoprostol 200 ug after 48 hours. The patient aborted with delivery of omphalopagus conjoined twins in vertex presentation. The induction to delivery interval from mifepristone was around 50 hours and from misoprostol was around 26 hours. Injection Pitocin 20 units with dilution was started. The placenta was delivered intact, and injection Methergine 0.2 mg IM was given. The cervix was traced for any tears. The patient was vitally stable and shifted to the ward. The procedure went smoothly, and the patient was discharged the next day. The pathology report confirmed the diagnosis of intact omphalopagus conjoined twins with a single gastrointestinal system, one placenta, four kidneys, two adrenal glands, two upper and two lower limbs, one umbilical cord, one umbilical artery, one umbilical vein, and one kidney that had spilled from the abdomen (Figure 3).

The final diagnosis was an omphalopagus conjoined twin with growth disparity between the two with the smaller twin having tetra-amelia and aqueduct stenosis. 

## Discussion

A definitive diagnosis of conjoined twin pregnancy is required in order to pick the appropriate treatment choice in terms of pregnancy termination or gestational continuation with surgical twin separation. Early pregnancy termination is a safer alternative since it suggests a lower emotional effect for the couple, which might be increased by the many multidisciplinary follow-ups necessary during the prenatal and postnatal period [11]. Gica et al. also concluded that early prenatal diagnosis is required since the perinatal management of conjoined twins presents several ethical problems [12]. An accurate diagnosis is crucial, and early detection in the first trimester allows doctors to appropriately advise the couple on whether to proceed with the pregnancy or for an early termination [12].

Ultrasonography is the investigation of choice, and a transvaginal volume probe can detect conjoined pregnancy as early as eight weeks of gestation. Prior to the invention of ultrasonography, such situations were never detected until a complication arose after birth or a cesarean section. In our situation, we were able to diagnose a mono-amniotic, monochorionic conjoined twin pregnancy by transabdominal and transvaginal ultrasound, providing the couple with a wide range of options. Although an early diagnosis is not always achievable, in this case, we made the diagnosis in the first trimester, and the couple could choose to terminate the pregnancy. The ultrasonographic results in our case were compatible with previously published diagnostic criteria for omphalopagus conjoined twins with tetra-amelia syndrome in the smaller twin.

Although a rare entity, clinicians and radiologists should have a high index of suspicion in all cases of twins and should actively look for conjoined twins and/or associated anomalies. Due to its rarity, the separation of conjoined twins poses a significant challenge to pediatric superspecialists. Although there is debate over the best time to have surgery, waiting a few months increases the likelihood of survival. When one twin puts the other's life in danger, early separation is likely to be required. Prior to the separation, a thorough anatomical study, careful surgical planning, and the twins' best health are required. Recent improvements in imaging methods for preoperative examinations offer a sufficient anatomical diagnosis and foretell the likelihood of separation. Results and survival rates have been improved, thanks to developments in anesthesia care and postoperative critical care.

## Conclusions

To provide an appropriate therapeutic option, a prompt diagnosis of conjoined twin pregnancy is crucial. Omphalopagus conjoined twins count for more than 10% of conjoined twin pregnancies, with the best chances of survival if successfully separated with an adequate multidisciplinary approach and preoperative planning for separation. In our case, the family was offered the opportunity to choose pregnancy termination, lessening the emotional anguish associated with this entity, thanks to the early diagnosis of the anomalous baby.
